# Peripheral ischemic limb necrosis in septic shock patient: A case report

**DOI:** 10.1097/MD.0000000000050692

**Published:** 2026-09-11

**Authors:** Ji Woong Ho, Young-Keun Lee

**Affiliations:** a Department of Orthopedic Surgery, Research Institute of Clinical Medicine of Jeonbuk National University – Biomedical Research Institute of Jeonbuk National University Hospital, Jeonju, Jeonbuk, Republic of Korea.

**Keywords:** amputation, disseminated intravascular coagulation, limb ischemia, septic shock, vasoconstrictor agents

## Abstract

**Rationale:**

Peripheral ischemic limb necrosis is a rare condition that progresses rapidly and aggressively, and eventually requires amputation. Disseminated intravascular coagulation (DIC) is a common cause of ischemic limb necrosis and vasopressors for maintaining blood pressure could be risk factors for ischemic limb necrosis. This case study aimed to identify the risk factors for ischemic limb necrosis in septic shock and determine the optimal time of surgical treatment for necrosis considering the progression of risk factors.

**Patient concerns:**

A 62-year-old male pig farmer visited the hospital complaining of dyspnea, general weakness, and skin color changes in the whole body, including both hands and feet. Initial vital signs were unstable.

**Diagnoses:**

The patient was diagnosed with sepsis and DIC based on the laboratory results. Blood cultures were positive for *S suis*. The skin lesions on both hands and feet were diagnosed as ischemic limb necrosis.

**Interventions:**

The patient was immediately treated with antibiotics, fluids, and vasopressors in the intensive care unit. Regular dressing was applied to the demarcated hands and feet. After 49 days, the left ring and small fingers and the right index, long, ring, and small fingers were amputated. Both toes were amputated simultaneously and debridement was performed on the left heel.

**Outcomes:**

No further necrosis was observed until postoperative 55 days. However, the left heel showed a soft tissue defect. Ultimately, a free ALT flap was created on the left heel by a plastic surgeon.

**Lessons:**

Physicians should consider peripheral ischemic limb necrosis in patients with septic shock presenting with DIC and treated with vasopressors. Surgical treatment should be performed only when necrosis is sufficiently demarcated considering the improvement in DIC and cessation periods of vasopressors without aggravating factors.

## 1. Introduction

In patients with septic shock, peripheral limb necrosis is often unexpectedly reported as a severe complication and is treated with multiple amputations.^[1,2]^ This could be explained by the effect of disseminated intravascular coagulation (DIC), which can induce occlusion of small vessels with thrombi and disruption of microcirculation in sepsis.^[1–3]^ Thus, DIC is regarded as a key factor explaining peripheral limb necrosis in septic shock.^[1,2,4,5]^

Patients with septic shock often have a hypo perfusion status. High-dose vasopressor therapy was used to maintain the mean arterial pressure (MAP).^[6]^ This appeared to be a risk factor for necrosis. However, the evidence is limited to a few cases and studies.^[4,6,7]^

Here, we present a case of septic shock with ischemic limb necrosis in a patient infected with *S sui*, a rapidly progressing and fatal zoonotic pathogen. In this case, we outline the risk factors for ischemic limb necrosis and optimal surgical management timing, considering the chronological progression of DIC, duration and doses of vasopressor support, and subsequent aggravation and cessation of skin lesion progression.

### 1.1. Consent

The patient signed an informed consent form for the publication of this case report and any accompanying images. The ethics committee of Jeonbuk National University Hospital waived the ethical approval for this study because it was a case report with fewer than 3 patients (CUH 2023–08–069).

## 2. Case presentation

A 62-year-old male patient was referred to our department because of necrotic changes in both the hands and feet. He initially complained of dyspnea, general weakness, and skin color changes in face and limbs and came to the emergency room. The patient had no medical history of cardiogenic disorders, peripheral arterial occlusion disorders, hypertension, chronic kidney disease, or diabetes mellitus. However, the patient also had a specific occupational history. He worked on pig farms to raise pigs. In the emergency room, his initial blood pressure was 60/39 mm Hg. His respiratory rate was 20/minutes and his peripheral saturation was 83%. The patient was alert. An emergency medical doctor suspected shock because of his unstable vital signs. During the inspection, a purpuric skin color change was found in the whole body, especially on his face, both hands, and both feet (Fig. 1).

**Figure 1.**
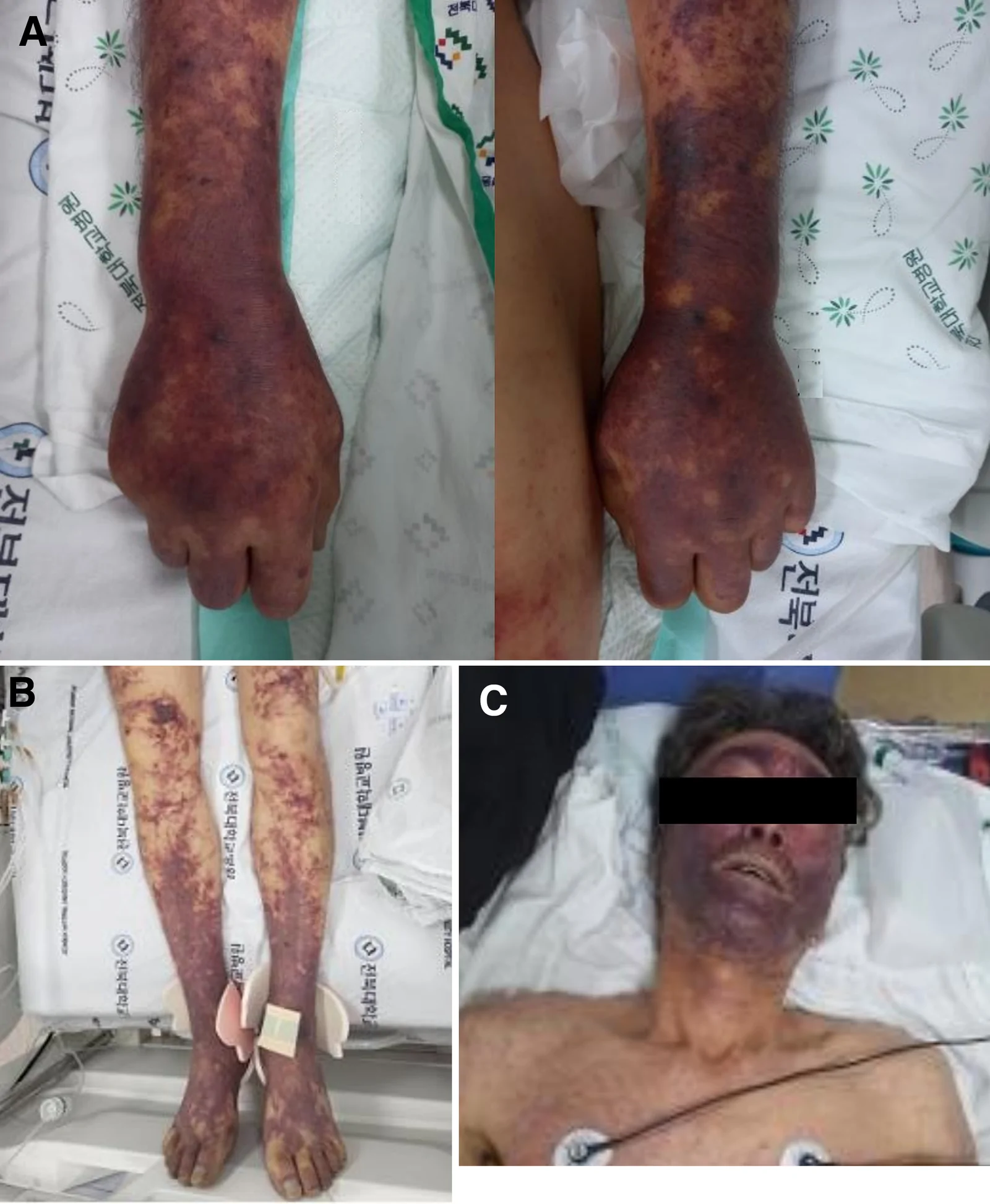
Gross photos of skin color changes of the patient. He first showed diffuse purpura-like color changes. (A) both hands, (B) feet, (C) face at emergency room.

His current status did not satisfy the quick Sequential Organ Failure Assessment (SOFA) score commonly used for suspected sepsis and septic shock. However, laboratory findings strongly suggested sepsis. Arterial blood gas analysis revealed lactic acidosis. Leukocytosis, thrombocytopenia, prothrombin time prolongation, and elevation of creatinine and D-dimer levels were also found in the laboratory tests. Blood cultures were positive for *S. suis* in the specimen (Table 1). He was diagnosed with sepsis and septic shock based on the International Society on Thrombosis and Hemostasis score. The patient score was 7 (Table 2).

**Table 1:** Initial laboratory results of the patient.

Variable*	Estimates(normal range)	Unit†
Lactate	9.0 (0.7–2.5)	mmol/L
WBC	13.41 (4.8 –10.8)	10^3/ul^
Platelet	24 (130–450)	10^3/ul^
PT	18.0 (9.2–12.6)	Sec
aPTT	77.2 (24.8–36.1)	Sec
D-dimer	92.437 (<0.49)	mg/L FEU
AST	550 (<40)	U/L
ALT	342 (<41)	U/L
Total bilirubin	3.74 (<1.2)	mg/dL
Direct bilirubin	3.23 (<0.3)	mg/dL
BUN	39 (8-23)	mg/dL
Cr	3.60 (0.7–1.7)	mg/dL
eGFR	17.1	mL/min/1.7

* aPTT = activated partial thromboplastin time, ALT = alanine amino transaminase, AST = aspartate amino transferase, BUN = blood urea nitrogen, Cr = creatinine, eGFR = estimated glomerular filtration rate, PT = prothrombin time, WBC = white blood cell.

† L, liter; 10^3,1000(cell count); ul, microliter; Sec, second; mg, milligram; FEU, fibrinogen equivalent unit; U, unit; dL, deciliter; min, minute.

**Table 2:** International Society on Thrombosis and Hemostasis (ISTH) score* and patient’s estimates.

Variable†	Range	Score	Patient’s estimate(Score)‡
Platelet count (10^3/ul)	>100,000	0	24,000 (2)
	50,000 ~ 100,000	1	
	<50,000	2	
Prolonged PT (sec)	<3	0	5.4 (1)
	3–6	1	
	>6	2	
Fibrinogen (mg/dL)	>100	0	88 (1)
	<100	1	
D-dimer (mg/L)	<0.5	0	92.437 (3)
	0.5~5	2	
	>5	3	

* ISTH score < 5 = not suggestive of overt disseminated intravascular coagulation (DIC); score ≥ 5 = compatible with overt DIC.

† 10^3,1000 (cell count); ul, microliter; PT, prothrombin time; sec, second; mg, milligram; dL, deciliter; L, liter.

‡ Estimated ISTH score of the patient is 7, which indicates overt disseminated intravascular coagulation (DIC).

He was immediately admitted to the intensive care unit (ICU) and treated with fluid resuscitation, antibiotics, intravenous immunoglobulin, and dexamethasone. Norepinephrine and vasopressin were infused for 3 days and 2 days each to maintain MAP and were gradually tapered. The weight-compensated peak dose of norepinephrine was 1.19 μg/kg/minutes. The weight-compensated peak dose of vasopressin was 0.0011 units/kg/minutes. DIC was also managed and prostaglandin E1 was administered. After admission to the ICU, the patient showed rapid necrotic changes in both the hands and feet. Necrosis in both hands and feet ceased to progress 13 days after the onset (Fig. 2A). Regular dressings were applied. Following recovery and discharge from the ICU, necrosis was gradually demarcated (Fig. 2B). No further disease progression was observed in this patient. The lesions were well demarcated 27 days after onset (Fig. 3).

**Figure 2.**
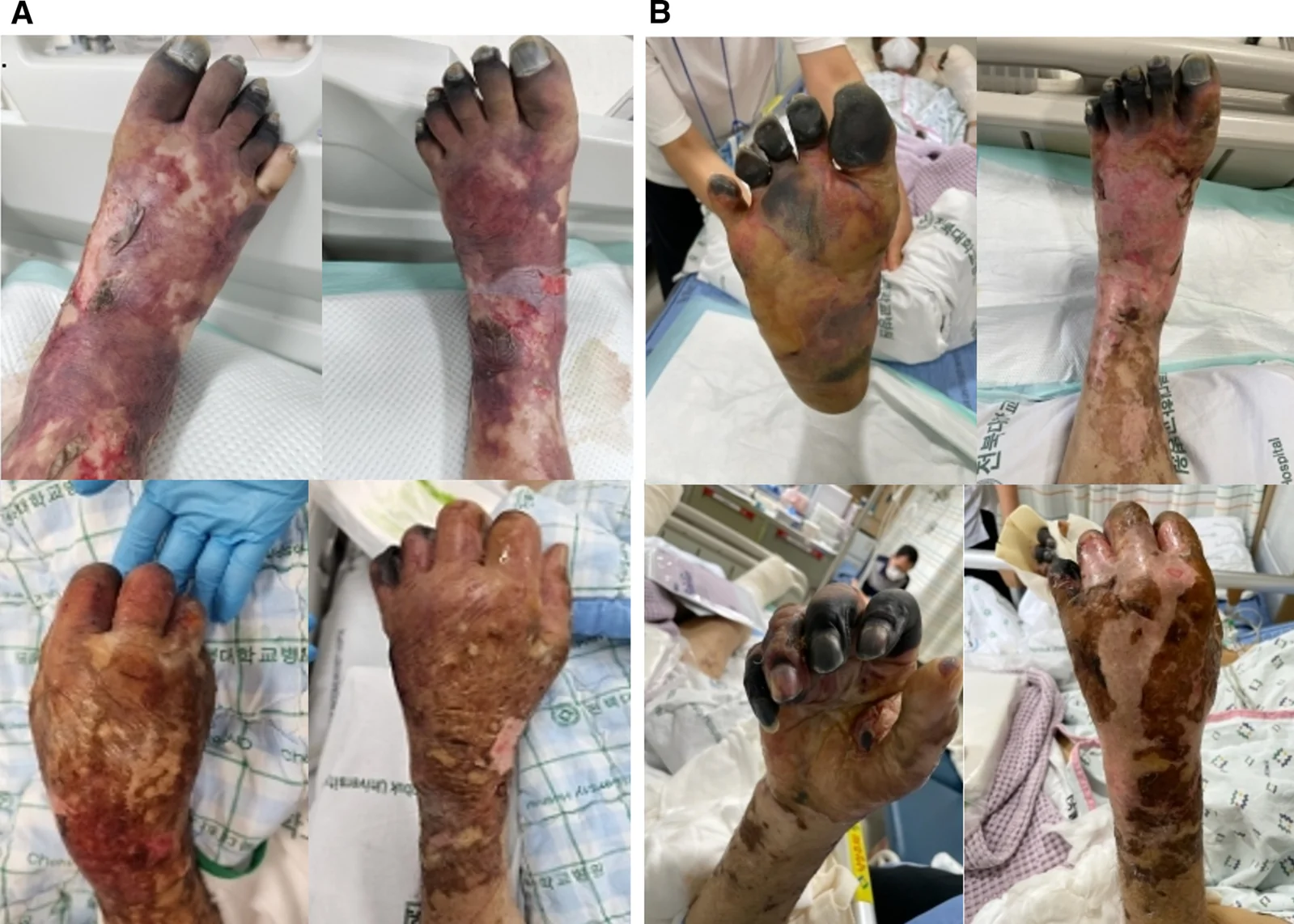
(A) Gross photos of skin lesions on both hands and feet at 13 days after onset. The range of lesions has stopped progressing compared to the initial skin lesions. (B) Gross photos of skin lesions at 19 days after onset show necrosis becoming gradually demarcated.

**Figure 3.**
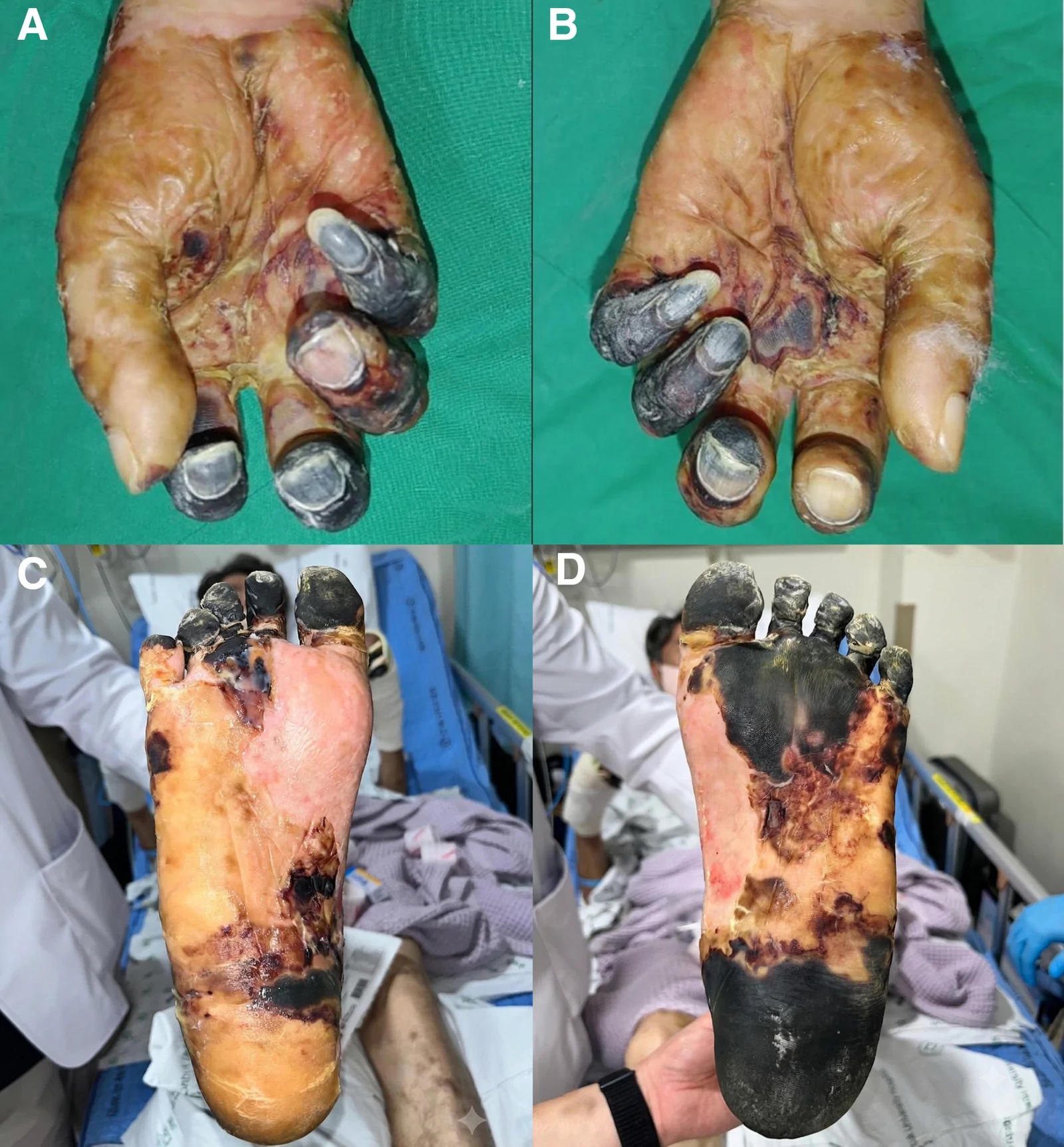
Gross photo of both hands at 27 days after onset. (A) Right hand showed RIF, RLF, RSF necrosis with demarcation extended to PIP joint, and RRF necrosis with extended to DIP joint. (B) Left hand showed LRF, LSF necrosis with demarcation extended to PIP joint and LLF necrosis extended to DIP joint. (C) Right foot showed necrosis with demarcation extended to toes and part of the sole in the foot. (D) Left foot showed necrosis with demarcation extended to toes, sole, and heel of the foot.(DIP = distal interphalangeal; LLF = left long finger, LRF=left ring finger; LSF=left small finger, PIP = proximal interphalangeal, RIF = right index finger, RLF = right long finger, RRF = right ring finger, RSF = right small finger).

We recommended that the patient undergo amputation surgery of the fingers 49 days after onset. We amputated his left ring finger and left small finger at the proximal phalanx level, right index finger and right long finger at the proximal phalanx level, right ring finger at the middle phalanx level, and right small finger at the proximal phalanx level (Fig. 4). Both toes were amputated simultaneously by foot and ankle orthopedic surgeons. Debridement was also performed for left heel necrosis.

**Figure 4.**
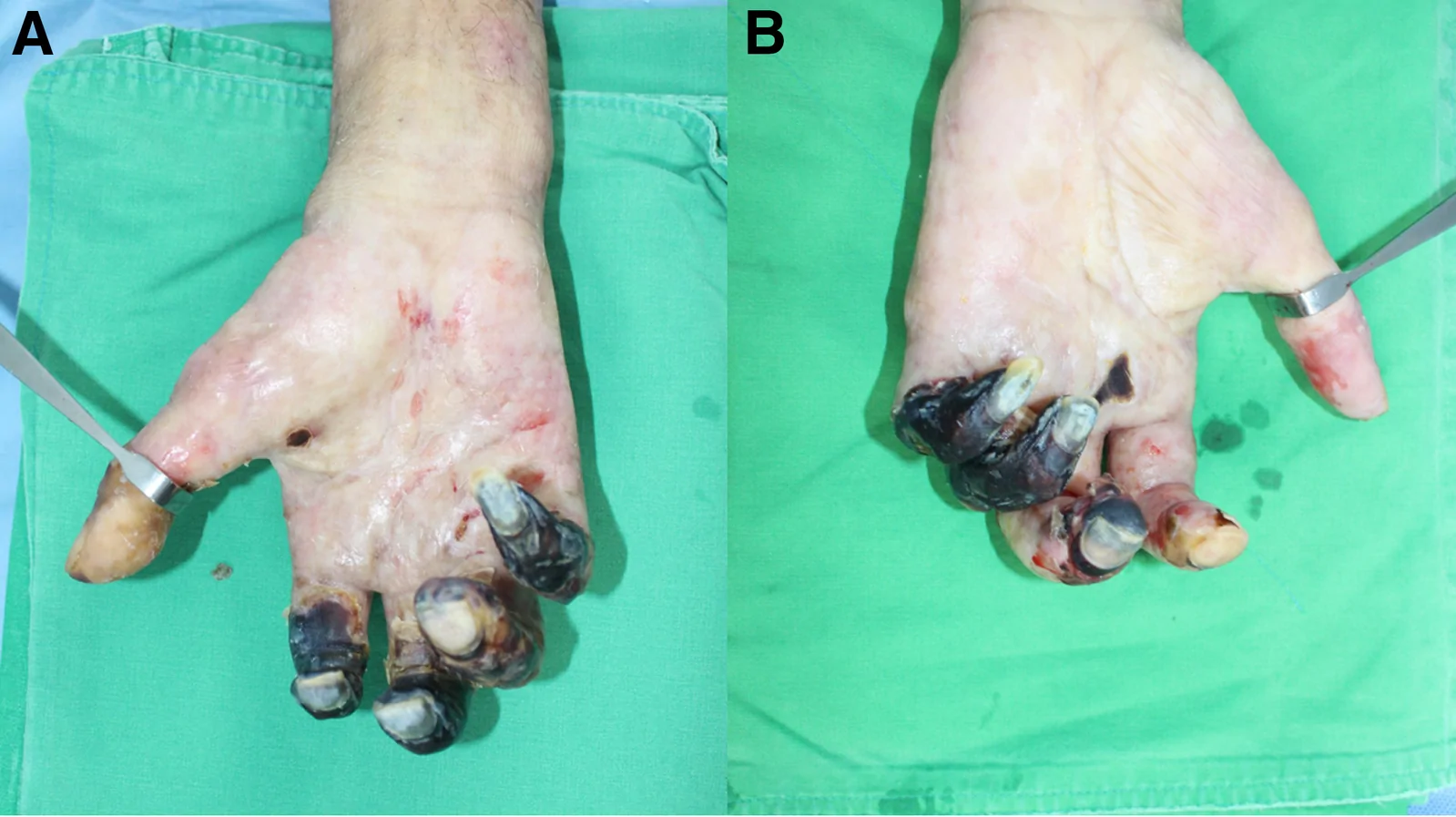
Intraoperative gross photo at 49days after onset. (A) We planned amputation of RIF and RLF at proximal phalanx level, RRF at middle phalanx level, and RSF at proximal phalanx level. **(B**) We planned amputation of LRF and LSF at proximal phalanx level. (LRF = left ring finger; LSF = left small finger; RIF = right index finger; RLF = right long finger; RRF = right ring finger; RSF = right small finger).

Postoperatively, neither finger showed additional necrotic changes. At 55 days after surgery, the wounds remained clean. However, both hands showed contracture and a limited range of motion. We trained the patient to perform passive flexion-extension exercises (Fig. 5). Neither toe showed any additional necrosis. However, the left heel lesion showed a soft tissue defect after debridement, and eventually, an anterolateral thigh free flap was created to cover the defect by a plastic surgeon (Fig. 6).

**Figure 5.**
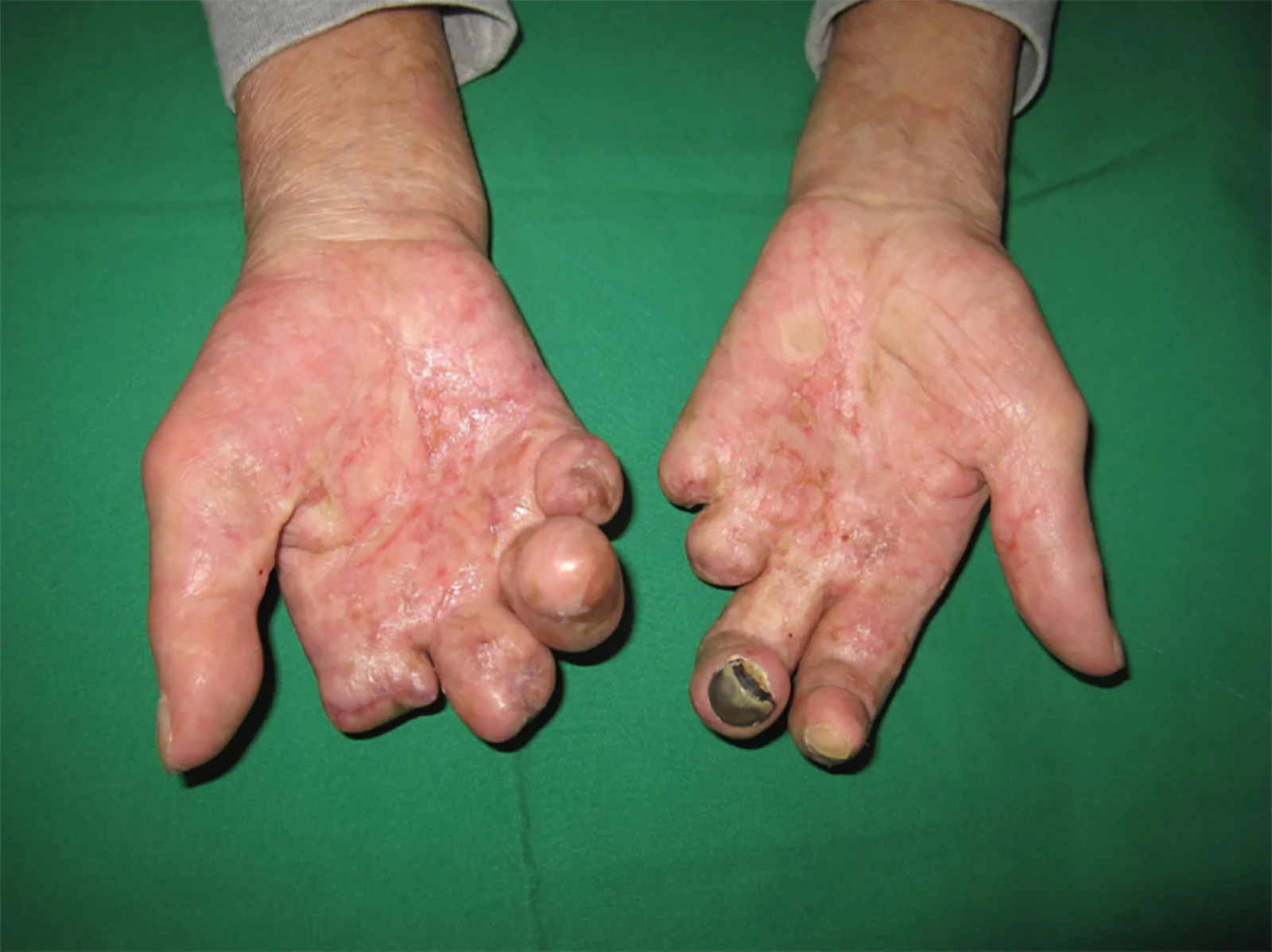
Gross photos of both hands at 55 days after amputation. There is no further necrotic change but both hands show contracture.

**Figure 6.**
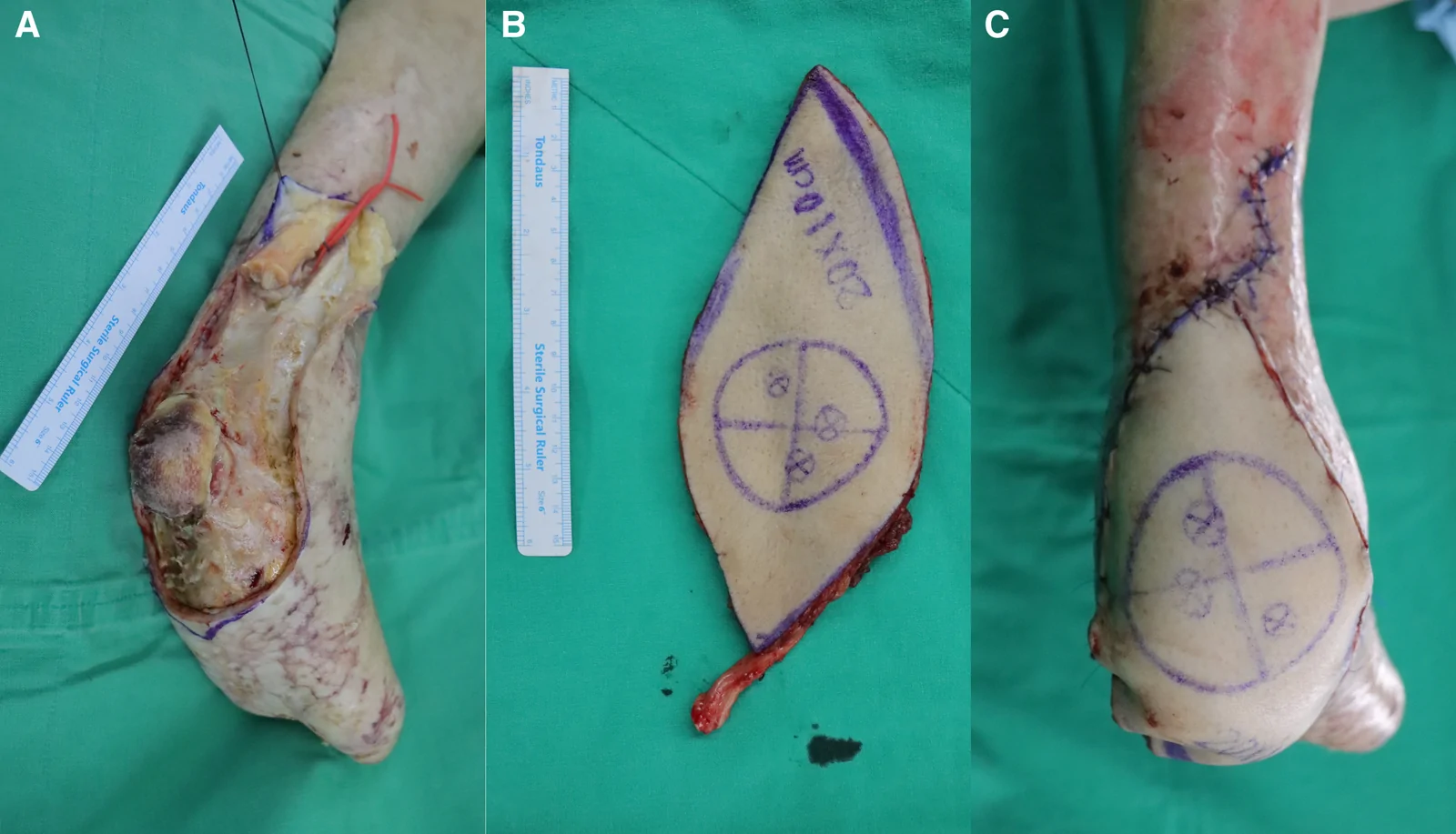
ALT free flap was performed to cover the softtissue defect of the left heel 3 months after debridement and amputation surgery. (A) Soft tissue defect of the left heel, (B) 20 × 10 cm sized ALT flap harvested from the ipsilateral thigh, and (C) free flap performed to cover the defect of the left heel.

## 3. Discussion

Peripheral limb necrosis is a condition in which the blood flow to the extremities decreases. Thus, tissues of the extremities, including bone, undergo ischemic changes.^[2]^ It can present as severe complications of various critically ill conditions affecting the peripheral vessels and the circulatory system, including shock (hypovolemic, cardiogenic or septic), cardiogenic thromboembolism, shock liver, peripheral arterial disease with atherosclerosis, DIC, and vasopressor therapy via peripheral vasoconstriction,^[1,2,6]^ although such complications are rare. DIC has been reported to be an important cause of ischemic limb necrosis.^[1,2,4]^ An early clinical finding that is suggestive of DIC is acrocyanosis. It can further develop into purpura fulminans and organ ischemic change finally.^[1,2]^

Several studies have shown that DIC is associated with ischemic limb necrosis under septic conditions. Iba et al^[8]^ explained that DIC is affected by vasoplegia and vascular endothelial injury in response to excessive blood protease activity caused by systemic inflammation. Vincent et al^[3]^ have also suggested that the vascular endothelium, platelets, and coagulation factors are damaged during sepsis, which can lead to an imbalance in the coagulation system, which at least develops DIC. They also explained that DIC could cause microthrombi in the vessels, resulting in limb necrosis and multiple organ failure. Thus, it is regarded as a key factor in explaining peripheral limb necrosis in septic shock.^[3,5,8]^

Septic shock with DIC should be managed with empirical antibiotics, fluid resuscitation, steroids, vasopressors, intravenous immunoglobulin, and prostaglandin E1.^[9,10]^ We should also consider the effect of high-dose vasopressors, such as norepinephrine, to maintain MAP in septic shock. Theoretically, high-dose vasopressors can constrict peripheral arterioles via α-adrenergic receptors and induce peripheral ischemic conditions. Kwon et al.^[6]^ retrospectively reviewed 36 patients with peripheral necrosis and 42 controls and suggested a statistical correlation between the dose and duration of vasopressors and necrosis. They suggested that the duration of vasopressor support was not significant; however, the weight-compensated peak dose of vasopressors was statistically significant. The range of the norepinephrine peak dose was 0to 0.894, and vasopressin was 0 to 0.014 in the patient group. However, they did not suggest cutoff values. Levy et.al.^[4]^ conducted a literature review and meta-analysis. They presented 3 studies that reported the relevance of vasopressors in peripheral limb necrosis.^[11–13]^ Among these 3 studies, 1 reported dose-dependent and duration-dependent correlations between vasopressors and necrosis.^[12]^ They reported that the mean dose of vasopressors (norepinephrine or epinephrine) was> 1μg/kg/minutes, and the mean duration of treatment was 84.7 hours. They presented possible effects of vasopressors on peripheral limb necrosis. Thus, there is no consensus on the use of vasopressors alone. It is considered to be a potential cause of ischemic limb necrosis in patients with septic shock.

The authors of this study noticed progression of DIC and necrosis in the present patient. The progression of necrosis rapidly exacerbated during the initial few days while the patient received care in the ICU. DIC estimated by the International Society on Thrombosis and Hemostasis score improved starting 9 days after onset. Skin lesions improved, and necrosis was demarcated 13 days after onset. Meanwhile, norepinephrine and vasopressin were injected intravenously during the initial 3 days. The peak dose of norepinephrine was higher than that reported in other studies. This progression seemed to have a strong correlation with the improvement in DIC and the relative correlation with vasopressor use. Thus, physicians should not only pay attention to the progression of DIC but also exercise caution when using vasopressors to treat septic shock in patients with peripheral limb necrosis.

Peripheral limb necrosis eventually requires surgical treatment, such as debridement and amputation, considering secondary infection and its underlying causes. Foead et al^[1]^ suggested that bullae in patients with peripheral limb necrosis should be treated to prevent secondary infection, and debridement of nonviable tissues should be performed as soon as the patient’s condition allows, even amputation. Warkentin et.al.^[2]^ suggested that surgical treatment may be required. However, early amputation should be avoided whenever possible because of the difficulty in distinguishing viable from necrotic tissues during the early phase of peripheral limb necrosis. Thus, we suggest that surgical treatment, including amputation, should be performed for peripheral limb necrosis only when the necrotic lesion is sufficiently demarcated without additional lesions. Physicians should also be convinced that the improvement of underlying causes such as DIC is clear and that sufficient periods of ceasing vasopressors are secured so that no aggravating factors remain in the patient.

This case report had several limitations. First, we could not check daily necrotic changes in the initial limb periods in the ICU, particularly during vasopressor treatment. Thus, we only studied the effects of vasopressors on necrosis using clinical data and medical records. Surgery was delayed until the patient’s general condition became tolerable to anesthesia and surgery. Thus, physicians should consider not only sufficient demarcation and improved causes, including DIC, but also the tolerable general condition of the patient when determining the proper surgery.

## 4. Conclusions

The authors suggest that physicians should consider peripheral ischemic limb necrosis in patients with septic shock presenting with DIC treated with vasopressors. Surgical treatment should be performed only when the necrotic lesion is sufficiently demarcated with confirmation of improvement in DIC and cessation periods of vasopressors without aggravating factors.

## Author contributions

**Data curation:** Ji Woong Ho.

**Formal analysis:** Ji Woong Ho, Young-Keun Lee.

**Methodology:** Ji Woong Ho, Young-Keun Lee.

**Conceptualization:** Young-Keun Lee.

**Supervision:** Young-Keun Lee.

**Writing – original draft:** Ji Woong Ho, Young-Keun Lee.

**Writing – review & editing:** Ji Woong Ho, Young-Keun Lee.
